# Flexible, durable, and anti-fouling maghemite copper oxide nanocomposite-based membrane with ultra-high flux and efficiency for oil-in-water emulsions separation

**DOI:** 10.1007/s11356-023-31240-x

**Published:** 2023-12-07

**Authors:** Mahmoud F. Mubarak, Hanaa Selim, Hamada B. Hawash, Mohamed Hemdan

**Affiliations:** 1 Department of Petroleum Application, Core Lab Analysis Center, Egyptian Petroleum Research Institute, P.B. 11727, Nasr City, Cairo Egypt; 2 Department of Analysis and Evaluation, Egyptian Petroleum Research Institute, Nasr City, 11727 Cairo Egypt; 3 Environmental Division, National Institute of Oceanography and Fisheries, NIOF, Cairo, Egypt; 4 School of Biotechnology, Badr University in Cairo (BUC), Badr City, 11829 Cairo Egypt

**Keywords:** Nanocomposite membrane, Poly(vinyl chloride), Maghemite copper oxide (MC), Separation efficiency, Durability, Antifouling

## Abstract

In this study, we developed a novel nanocomposite-based membrane using maghemite copper oxide (MC) to enhance the separation efficiency of poly(vinyl chloride) (PVC) membranes for oil-in-water emulsions. The MC nanocomposite was synthesized using a co-precipitation method and incorporated into a PVC matrix by casting. The resulting nanocomposite-based membrane demonstrated a high degree of crystallinity and well-dispersed nanostructure, as confirmed by TEM, SEM, XRD, and FT-IR analyses. The performance of the membrane was evaluated in terms of water flux, solute rejection, and anti-fouling properties. The pinnacle of performance was unequivocally reached with a solution dosage of 50 mL, a solution concentration of 100 mg L^−1^, and a pump pressure of 2 bar, ensuring that every facet of the membrane’s potential was fully harnessed. The new fabricated membrane exhibited superior efficiency for oil–water separation, with a rejection rate of 98% and an ultra-high flux of 0.102 L/m^2^ h compared to pure PVC membranes with about 90% rejection rate and an ultra-high flux of 0.085 L/m^2^ h. Furthermore, meticulous contact angle measurements revealed that the PMC nanocomposite membrane exhibited markedly lower contact angles (65° with water, 50° with ethanol, and 25° with hexane) compared to PVC membranes. This substantial reduction, transitioning from 85 to 65° with water, 65 to 50° with ethanol, and 45 to 25° with hexane for pure PVC membranes, underscores the profound enhancement in hydrophilicity attributed to the heightened nanoparticle content. Importantly, the rejection efficiency remained stable over five cycles, indicating excellent anti-fouling and cycling stability. The results highlight the potential of the maghemite copper oxide nanocomposite-based PVC membrane as a promising material for effective oil-in-water emulsion separation. This development opens up new possibilities for more flexible, durable, and anti-fouling membranes, making them ideal candidates for potential applications in separation technology. The presented findings provide valuable information for the advancement of membrane technology and its utilization in various industries, addressing the pressing challenge of oil-induced water pollution and promoting environmental sustainability.

**Graphical Abstract:**

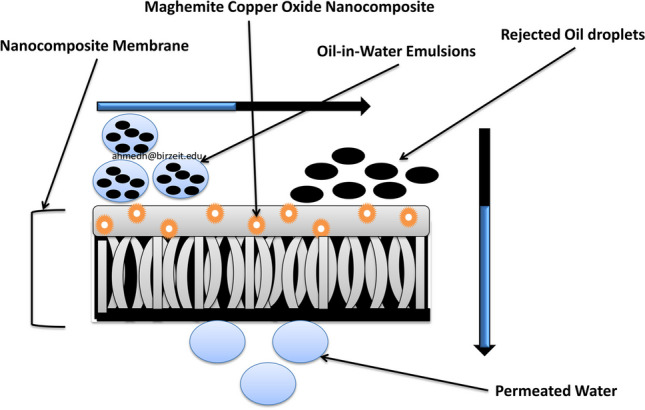

## Introduction

Oil-in-water emulsion separation is a significant and growing challenge in various industries, encompassing sectors like oil, gas, and food production. The primary driver behind this challenge is the escalating concern over water pollution and the diminishing availability of freshwater resources (Dhandhi et al. 2022; Tian et al. 2022; José et al. 2023). Conventional methods for separating oil from water in emulsions often face critical limitations, including low separation efficiency and inadequate durability. These limitations, in turn, contribute to substantial environmental and economic consequences (Moghaddasi et al. 2020). To confront these challenges and advocate for the conservation of water resources, it is crucial to develop effective and sustainable techniques for treating oily wastewater (Li et al. 2021; Chowdhury et al. 2022).

Numerous techniques have been explored to treat oily wastewater, including advanced oxidation processes (AOP) (Golshenas et al. 2020), flotation (Ogunbiyi and Liu 2023)(Silva et al. 2019), coagulation(You et al. 2018), ultrasonic separation (Atehortúa et al. 2019), adsorption (Yu et al. 2021), photocatalysis (Yan et al. 2020), skimming (Feng et al. 2020), biological treatment (Camarillo and Stringfellow 2018), chemical de-emulsification, and air flotation (Wang et al. 2022). However, these methods often suffer from drawbacks such as high energy consumption, secondary pollution generation, low efficiency, high costs, large equipment size, and recontamination. As a result, there is a pressing need to develop more effective and sustainable separation technologies (Adetunji and Olaniran 2021).

Membrane technology has emerged as a promising solution for various separation processes due to its versatility and efficiency(Ezugbe and Rathilal 2020; Obotey Ezugbe and Rathilal 2020; Bera et al. 2021; Yadav et al. 2022). Among the different polymer materials explored for membrane fabrication, poly(vinyl chloride) (PVC) is a widely used commercial plastic known for its broad range of applications (Safarpour et al. 2021; Ahmad et al. 2023). Although PVC membranes have shown potential for oil-in-water emulsion separation, they have received less attention in research compared to other materials. Various studies have highlighted potential applications of PVC membranes. For instance, researchers have found that incorporating polyvinyl butyral (PVB) into PVC membranes enhances their characteristics and performance (Shen et al. 2005; Zheng et al. 2018; Safarpour et al. 2021). Additionally, the use of polyvinylpyrrolidone (PVP) and polyethylene glycol (PEG) has been explored for improving PVC membrane properties (Wongchitphimon et al. 2011; Gebru and Das 2017; Kahrs and Schwellenbach 2020).

The integration of nanoparticles into membrane technology has garnered substantial attention in recent years, primarily due to their transformative potential across various applications, notably in the realm of oil–water emulsion separation (Yi et al. 2013; Sinha Ray et al. 2020; Nain et al. 2022). Magnetic nanoparticles (MNPs) stand out owing to their unique physicochemical characteristics and exceptional biocompatibility, making them highly promising as additives in different types of membranes, such as polysulfone (PS), polyvinylidene fluoride (PVDF), and polyethersulfone, all widely employed for the treatment of oily wastewater (Heidi Lynn et al. 2012; Rashdan et al. 2013; De Sitter et al. 2014; Homayoonfal et al. 2014; Barroso-Solares et al. 2018; Ramazanov et al. 2020; Mehrnia et al. 2021; Mirzaei et al. 2021; Koyuncu et al. 2022). The incorporation of MNPs into these membranes has yielded tangible benefits, particularly in terms of bolstering their antifouling properties, thereby enhancing overall performance. This advancement holds particular significance in the context of oil–water emulsions, where fouling can severely impede the efficiency of separation processes. Consequently, the utilization of MNPs within membrane technology holds immense potential in elevating the efficiency and sustainability of oil–water emulsion separation processes(Kong et al. 2022).

Our study introduces a novel approach by utilizing maghemite copper oxide (MC) nanocomposites to improve PVC membranes. MC, synthesized through a co-precipitation method, exhibits distinctive physicochemical characteristics and exceptional biocompatibility. The incorporation of MC nanocomposite into PVC matrices is expected to significantly enhance membrane antifouling properties and separation efficiency (Demirel et al. 2017; Agboola et al. 2021). MC nanoparticles offer a substantial surface area and large pore volumes, creating additional adsorption active sites for oil droplets. Moreover, the catalytic potential of copper oxide nanoparticles within MC facilitates the efficient breakdown of oil droplets into smaller, more readily separable components. This innovative approach holds promise for advancing membrane technology in various applications (Gawande et al. 2016; Elmobarak and Almomani 2021). PVC membranes were chosen as the focus of our investigation due to their widespread use and compatibility with various industrial and environmental settings. PVC offers a cost-effective, scalable, and readily available membrane material, making it an ideal candidate for real-world applications. This choice enables us to address the immediate and practical need for improving the efficiency of PVC membranes in various industries, including wastewater treatment and oil–water separation (Safarpour et al. 2021) (Ahmad and Guria 2022).

In this study, we aim to fabricate and characterize the maghemite copper oxide nanocomposite-based PVC membrane. We will investigate its structure and properties using various analytical techniques such as transmission electron microscopy (TEM), scanning electron microscopy (SEM), X-ray diffraction (XRD), and Fourier transform infrared (FT-IR) spectroscopy. The membrane’s performance will be evaluated in terms of water flux, solutes rejection, and anti-fouling properties. This research aims to provide valuable insights into the development of more flexible, durable, and anti-fouling membranes, addressing the pressing challenge of oil-induced water pollution and promoting environmental sustainability. This study aligns with the need for sustainable solutions in the face of increasing environmental challenges, making it an essential contribution to the field of membrane-based emulsion separation.

## Experimental

### Materials

In this research, we exclusively utilized reagent-grade substances, all of which were procured from Sigma-Aldrich in Cairo, Egypt. These substances included ferrous sulfate heptahydrate (FeSO_4_.7H_2_O), copper sulfate pentahydrate (CuSO_4_.5H_2_O), polyvinyl chloride (PVC), sodium dodecylbenzene sulfonate (SDBS), dimethylformamide (DMF), sodium hydroxide (NaOH) powder, and NH_4_Cl (ammonium chloride). All of these materials were sourced from Merck KGaA in Frankfurt, Germany. To prepare our solutions, we employed fresh double deionized water (DDW). It is noteworthy that the chosen substances had a purity level of 99% and met the standards for analytical-grade materials.

### Preparation of maghemite seeds (M)

Maghemite seeds were synthesized through the following procedure: Initially, ferrous sulfate heptahydrate (FeSO_4_.7H_2_O) was dissolved in 100 mL of double deionized water (DDW) with continuous stirring until complete dissolution. Simultaneously, a 0.1 M sodium hydroxide (NaOH) solution was prepared by dissolving 10 mL of NaOH in 10 mL of DDW. The NaOH solution was gradually added to the ferrous sulfate solution at a rate of 1 mL per 10 min. The resulting mixture was then heated to 70 °C and maintained at this temperature for approximately 2 h. Afterward, the mixture was allowed to cool down to room temperature and was stabilized by the addition of 10 mL of NH_4_Cl solution (0.1 M). The resulting black maghemite seeds were thoroughly washed with DDW multiple times until reaching a neutral pH. Finally, the maghemite seeds were separated through centrifugation and subsequently dried overnight in an electric oven, yielding the maghemite seeds that were subsequently used in the subsequent steps of the study.

### Synthesis of maghemite copper oxide (MC) nanocomposite

In a quest for scientific excellence, the maghemite copper oxide (MC) nanocomposite was meticulously synthesized. The maghemite seeds, carefully obtained from the previous step, were dispersed in 100 mL of double deionized water (DDW). Meanwhile, in a separate process, copper sulfate pentahydrate (CuSO_4_.5H_2_O) was dissolved in 20 mL of DDW at a precise 2:1 ratio. The copper sulfate solution was then seamlessly combined with the maghemite seed dispersion. Following this harmonious union, the mixture was gently stirred for 2 h at room temperature, allowing for a smooth interaction of the components. Subsequently, a delicate rise in temperature to 70 °C continued the reaction for an additional 3 h, bringing the synthesis to its pinnacle. The result of this carefully orchestrated alchemy was the creation of the maghemite copper oxide (MC) nanocomposite. Its unique attributes and vast potential make it an intriguing prospect for diverse applications, leaving researchers and enthusiasts captivated by the possibilities that lie ahead.

### Membrane (PMC) preparation

#### Preparation of PVC support

The poly(vinyl chloride) (PVC) support for casting the maghemite copper oxide nanocomposite (MC) was prepared using a phase inversion technique. First, 500 g of PVC was dissolved in 50 mL of dimethylformamide (DMF) under continuous stirring until a homogeneous solution was obtained. The dissolution process was carried out at room temperature to ensure complete mixing. Next, the MC nanocomposite was incorporated into the PVC solution at a ratio of 1:10 (MC:PVC) and stirred for 30 min to ensure proper dispersion of the nanocomposite within the PVC matrix. To improve the compatibility between MC and PVC, 50 g of sodium dodecylbenzene sulfonate (SDBS) was added to the mixture and stirred for an additional 30 min.

#### Casting and drying of nanocomposite membrane

**Scheme 1.**
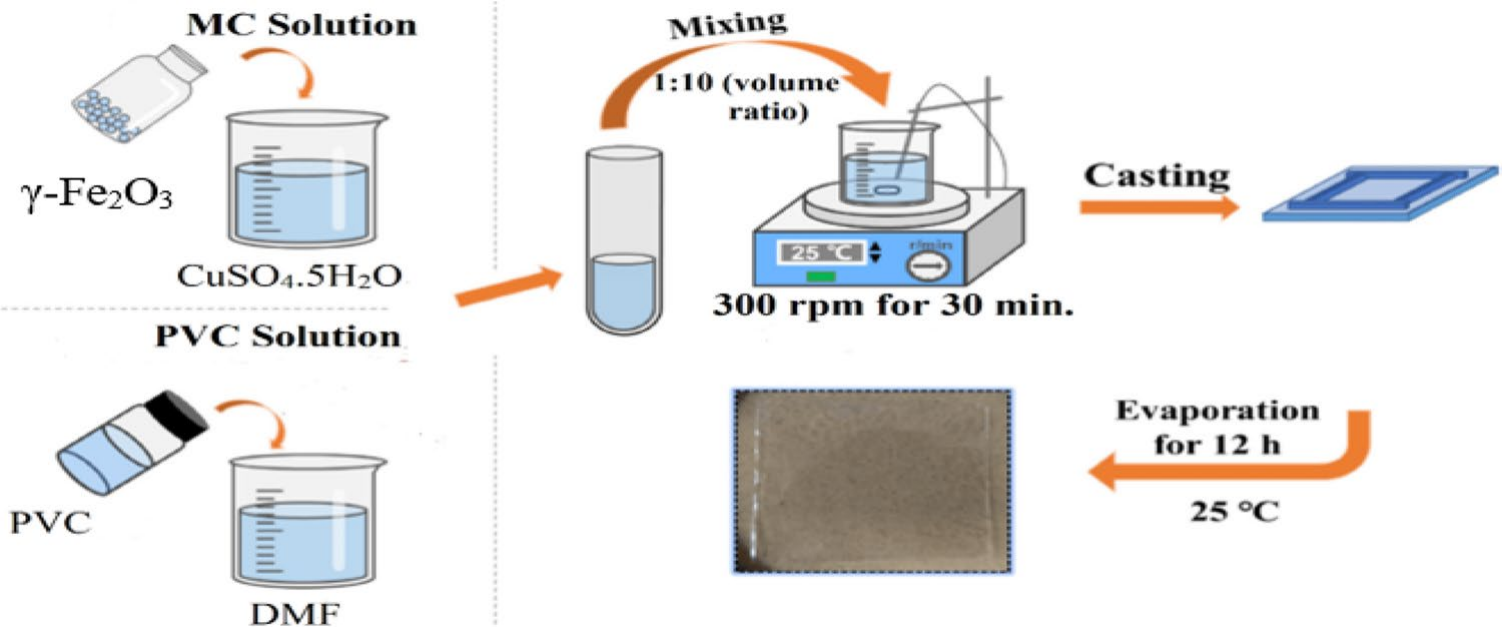
Schematic representation for membrane preparation

After the preparation of the PVC solution containing the MC nanocomposite as shown in Scheme 1, the casting procedure was initiated. A glass plate was used as the substrate for casting the membrane. Prior to casting, the glass plate was cleaned thoroughly and dried to ensure a clean and smooth surface. The PMC nanocomposite solution was poured onto the glass plate, and a casting knife was used to control the thickness of the membrane. A cast film with a thickness of approximately 0.6 mm was obtained by adjusting the knife gap. To remove any trapped air bubbles, the cast film was subjected to vacuum treatment in a vacuum oven for 12 h.

After the vacuum treatment, the nanocomposite membrane was transferred to a coagulation bath containing double deionized water (DDW). The membrane was allowed to coagulate in the bath for 24 h to ensure proper phase separation and solvent removal. During this process, the nanocomposite membrane formed a porous structure due to the phase inversion of PVC and the precipitation of PMC nanocomposite on the surface. Following the coagulation process, the nanocomposite membrane was washed thoroughly with DDW to eliminate any residual solvent and unbound nanocomposite particles. The washing process was repeated multiple times until a neutral pH was achieved, indicating the removal of any remaining chemicals or impurities.

### Characterization of membrane

The membranes crafted in the laboratory were subjected to a comprehensive and artistic examination, aiming to unravel their hidden mysteries. The esteemed high-resolution transmission electron microscope (HRTEM) and scanning electron microscope (SEM) gracefully took the stage, operating at an accelerating voltage of 200 kV, skillfully capturing intricate details of the membranes’ crystal structure, size, and surface morphology. To illuminate the enigmatic phases concealed within the samples, X-ray diffraction (XRD) showcased its brilliance with a state-of-the-art diffractometer (Philips 1830). Bathed in the radiance of CuKα radiation (*λ* = 1.5418 Å) at 40 kV and 40 mA, the membranes’ secrets were gracefully unveiled, as the 2θ range from 4 to 80° cast its intriguing spotlight on their inner architecture. The enchanting melody of Fourier transform infrared (FT-IR) spectroscopy graced the scene, conducted with the elegant model Spectrum One instrument (Perkin Elmer, USA). The symphony of wavenumbers from 400 to 4000 cm^−1^ was deftly explored, allowing the identification of the harmonious functional groups present in the membranes. The hydrophilic/hydrophobic characteristics of the membranes were evaluated by measuring the contact angle between the membrane surface and water droplet using a contact angle goniometer (ZAM104-B, Zolalan Co., Iran) and analyzed by Cooling Tech software. Through this captivating performance of scientific artistry, the membranes unveiled their essence, leaving researchers and visionaries enchanted by their unique qualities and the endless possibilities they hold.

## Membrane properties

### Evaluation of separation performance of the synthesized membrane

The evaluation of the synthesized membrane’s separation performance was conducted with utmost precision, employing established methodologies as described by Cai et al. (2017). To gauge its efficacy, we meticulously examined the retention of oil within the oil-in-water emulsion, employing a well-established equation, expressed as follows:

$$R=\frac{{C}_{o}-{C}_{p}}{{C}_{o}}*100 \%$$

In this equation, *R* represents the percentage of separation efficacy achieved by the membrane, while *C*_*o*_ and *C*_*p*_correspond to the concentrations of the original oil-in-water and the collected oil, respectively. The concentrations *C*_*o*_ and *C*_*p*_ were determined employing the advanced Water Detective 3 multiparameter spectrum water quality analyzer, ensuring accurate and reliable measurements.

To quantitatively assess the membrane’s performance, the coveted flux (*J*) of the membrane for a given emulsion was deduced through the following equation (Cai et al. 2017):

$$J=\frac{\mathrm{V}}{A*t*\mathrm{\Delta P}}$$

In this expression, *J* stands for the membrane flux, expressed in L m^−2^ h^−1^ bar^−1^. The symbol *A* (m^2^) signifies the membrane’s effective filtration area, *V* (L) represents the volume of permeate obtained, and Δ*P* represents the applied external pressure in bar. The time of separation *t* (h) is an essential parameter in this equation.

With unwavering dedication, we conducted multiple trials, consistently casting a fixed quantity of emulsion into the filter. To ensure the robustness of our findings, we diligently examined six samples for each system, deriving an average value to validate the membrane’s prowess. The culmination of our diligent efforts revealed the synthesized membrane’s exceptional capability to efficiently separate oil-in-water emulsions. These findings, backed by accurate data and analysis, provide profound insights into the membrane’s promise for practical implementation across diverse environmental and industrial applications.

## Results and discussion

### Characterization of the membrane

#### Nanocomposite structure revealed by transmission electron microscopy (TEM)

**Fig. 1:**
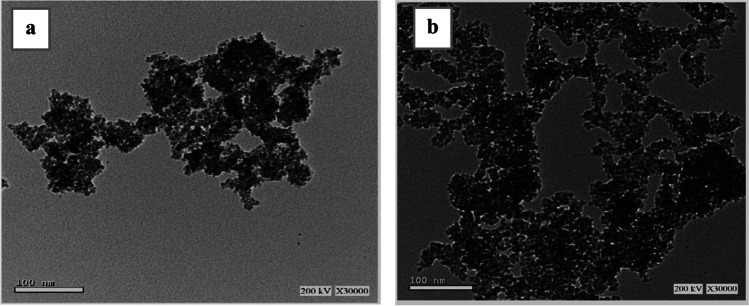
TEM images of **a** maghemite copper oxide (MC) nanocomposite and **b** gamma magnetite

The TEM image of the maghemite copper oxide (MC) nanocomposite reveals a distinct cluster of copper oxide molecules intricately arranged in a hexagonal pattern, each of these molecules measuring approximately 100 nm in size as shown in Fig. 1a. Concurrently, the image exhibits certain dark spots, likely attributed to impurities or defects within the nanocomposite structure. This hexagonal pattern in the copper oxide molecules can be attributed to their crystal structure, even though copper oxide inherently adopts a cubic crystal form. The unique arrangement arises from the stacking of unit cells, forming this captivating hexagonal configuration. Notably, a similar hexagonal pattern is also evident in the TEM image of the gamma magnetite nanocomposite as shown in Fig. 1b. The MC nanocomposite holds significant promise, given its likely possession of various intriguing properties, including elevated electrical conductivity and magnetic permeability. The hexagonal pattern of the copper oxide molecules is due to their crystal structure. Copper oxide is a cubic crystal, but the unit cells are stacked in a way that forms a hexagonal pattern. The hexagonal pattern is also visible in the TEM image of the gamma magnetite nanocomposite. The MC nanocomposite is likely to have a number of interesting properties, such as high electrical conductivity and magnetic permeability.

#### Surface morphology probed by scanning electron microscopy (SEM)

**Fig. 2:**
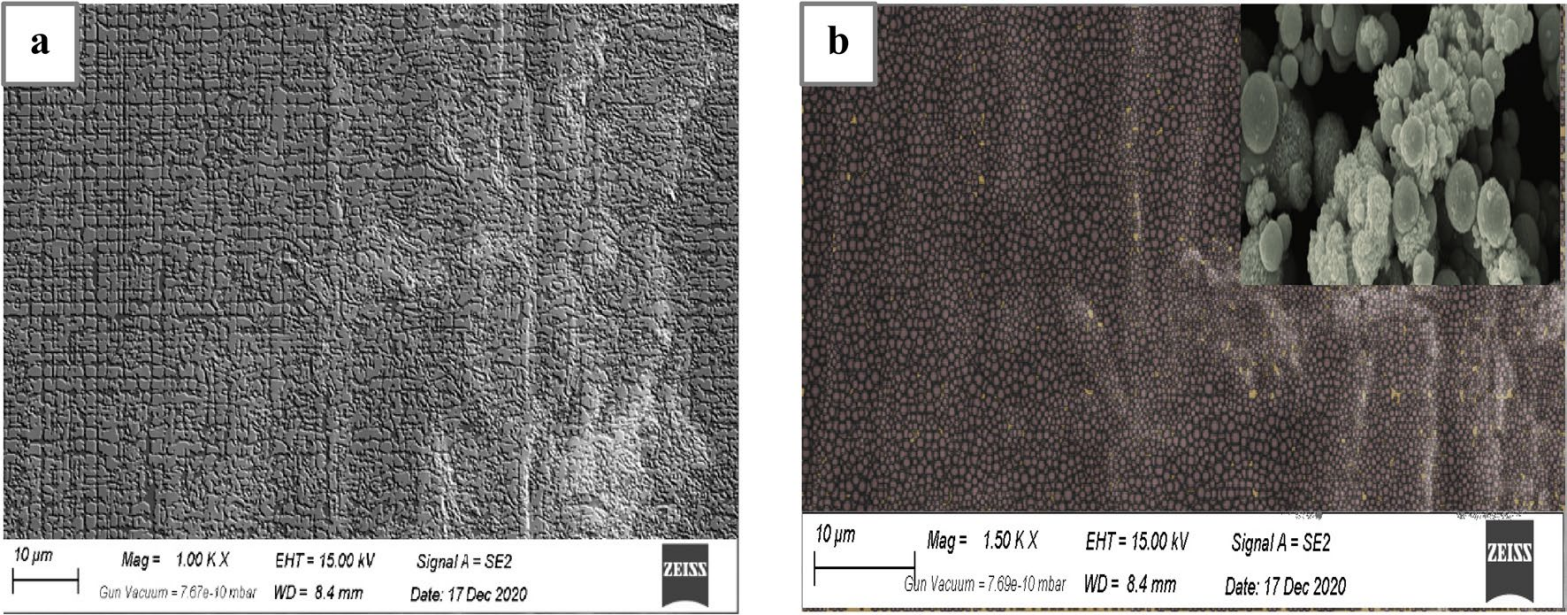
SEM images of: **a** PMC nanocomposites membrane and **b** PVC blank membrane

Scanning electron microscopy (SEM) analysis was employed to investigate the surface morphology of the composite membrane. In Fig. 2a, the SEM image of the PMC nanocomposite membrane reveals a more complex and rough surface morphology compared to the PVC blank membrane. This surface roughness is a result of the maghemite copper oxide nanoparticles present, leading to the formation of roughness, bumps, and protrusions on the membrane surface. Conversely, Fig. 2b depicts the PVC blank membrane, which exhibits a smoother and more uniform surface without any prominent features or defects. Moreover, the presence of nanoparticles in the PMC nanocomposite membrane may influence the pore size distribution, potentially affecting the overall membrane porosity. SEM analysis provides valuable insights into the surface characteristics, pore structure, and interfacial interactions within the PMC nanocomposite membrane and the PVC blank membrane, contributing to a comprehensive understanding of their properties and performance.

#### Crystal structure confirmed by X-ray diffraction (XRD) analysis

**Fig. 3:**
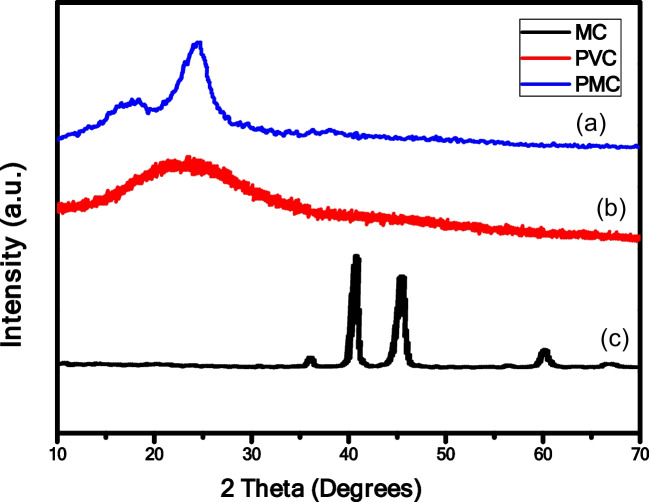
XRD pattern of: **a** PMC nanocomposites membrane, **b** PVC, and **c** MC

X-ray diffraction (XRD) analysis was employed to confirm the crystallinity of the synthesized PMC, PVC, and MC NPs. As depicted in Fig. 3a, b, c, the XRD pattern of MC NPs exhibits characteristic peaks at 2θ values of 35.97°, 40.49°, 45.32°, 60.12°, and 66.67° corresponding to pure γ-Fe_2_O_3_. Furthermore, peaks observed at 2θ values of 35.97°, 45.32°, 60.12°, 66.67°, 74.06°, and 79.18° correspond to CuO, confirming the successful synthesis of maghemite copper oxide nanocomposites (Li et al. 2010). In contrast, the XRD spectrum of the PVC blank membrane may show no distinct diffraction peaks or very weak peaks due to the amorphous nature of the PVC matrix. Interestingly, the PMC nanocomposite membrane displays additional diffraction peaks at angles of 17.31°, 24.23°, 35.33°, and 37.52°, attributed to the presence of maghemite copper oxide nanoparticles within the PVC matrix. Furthermore, the original characteristic peaks of maghemite copper oxide nanoparticles (MC NPs) may undergo reduction, slight shifting, or disappearance in the composite pattern due to the insertion of MC NPs. These XRD results confirm the successful fabrication of the composite membranes. Moreover, the PMC nanocomposite membrane exhibits a higher degree of crystallinity compared to the PVC blank membrane, likely due to the presence of maghemite copper oxide nanoparticles inducing crystallization of the PVC matrix.

#### Molecular structure characterization using Fourier transform infrared (FT-IR) spectroscopy

**Fig. 4:**
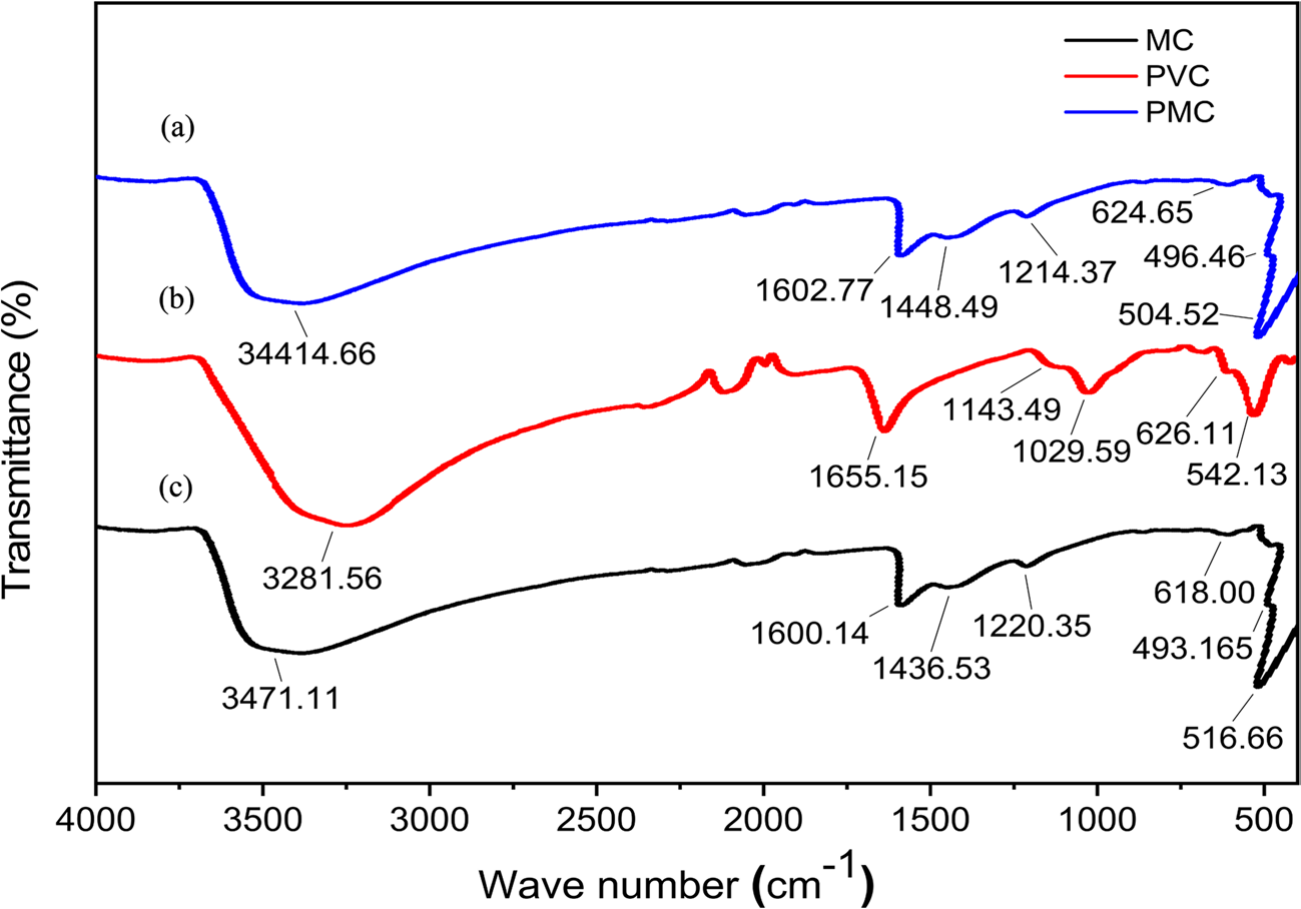
FT-IR spectra of: **a** PMC, **b** PVC, and **c** MC

Fourier transform infrared (FT-IR) spectroscopy was employed to characterize the molecular structure of the materials. As shown in Fig. 4a, b, c, the FT-IR spectra provide valuable information about the functional groups and chemical bonds present in MC NPs, PVC, and the PMC composites. The peaks observed at around 516.66 and 618.00 cm^−1^ in the FT-IR spectrum of MC NPs (Fig. 4c) are ascribed to Fe–O stretching vibrations, indicating the presence of Fe_2_O_3_ (Lesiak et al. 2019). Additionally, peaks at 1220.35 and 1436.53 cm^−1^ correspond to Cu–O and Cu–OH, respectively, further confirming the formation of maghemite copper oxide. The broader peak observed at 3471.16 cm^−1^ is attributed to the stretching vibrations of OH groups on the MC surface. By comparing the FT-IR spectra of the PMC nanocomposite membrane and the PVC blank membrane, changes in the molecular structure resulting from the incorporation of MC NPs into the PVC matrix are evident. In Fig. 4b, the FT-IR spectrum of the PVC blank membrane typically exhibits characteristic peaks at approximately 2960 and 2845 cm^−1^, corresponding to the C–H stretching of the polymer backbone. Other peaks at 626.11 cm^−1^ and 1029.59 cm^−1^ correspond to C–Cl and C–C stretching, respectively. Additionally, the peak around 1143.49 cm^−1^ indicates the C–O stretching vibrations of PVC. The presence of maghemite copper oxide nanoparticles in the PMC nanocomposite membrane introduces an additional peak in the range of 400–800 cm^−1^ (Fig. 4a). These FT-IR results offer valuable insights into the molecular structure, functional groups, and chemical interactions within the PMC nanocomposite membrane and the PVC blank membrane, further contributing to our understanding of their properties and performance.

In conclusion, the PMC nanocomposite membrane demonstrates significant improvements in separation performance compared to the PVC blank membrane. The incorporation of maghemite copper oxide nanoparticles enhances hydrophilicity, provides a nanostructured morphology, increases membrane stability, and catalyzes emulsion breakup. The study paves the way for the development of advanced nanocomposite membranes with broad applications in various industries and environmental sectors.

### Investigation of various operational parameters

The influence of several key parameters on the rejection rate and flux of the PMC membrane nanocomposite and PVC blank membrane was comprehensively investigated. These parameters included solution dosage (10, 20, 30, 40, 50, 60, and 70 mL), solution concentration (10, 30, 40, 60, and 100 mg L^−1^), and pump pressure (0.5, 1.0, 1.5, 2.0, and 2.5 bar). To assess the membrane’s performance under various conditions, a single-factor experimental design was employed, ensuring a reliable and accessible separation process.

#### Impact of solution dosage

**Fig. 5:**
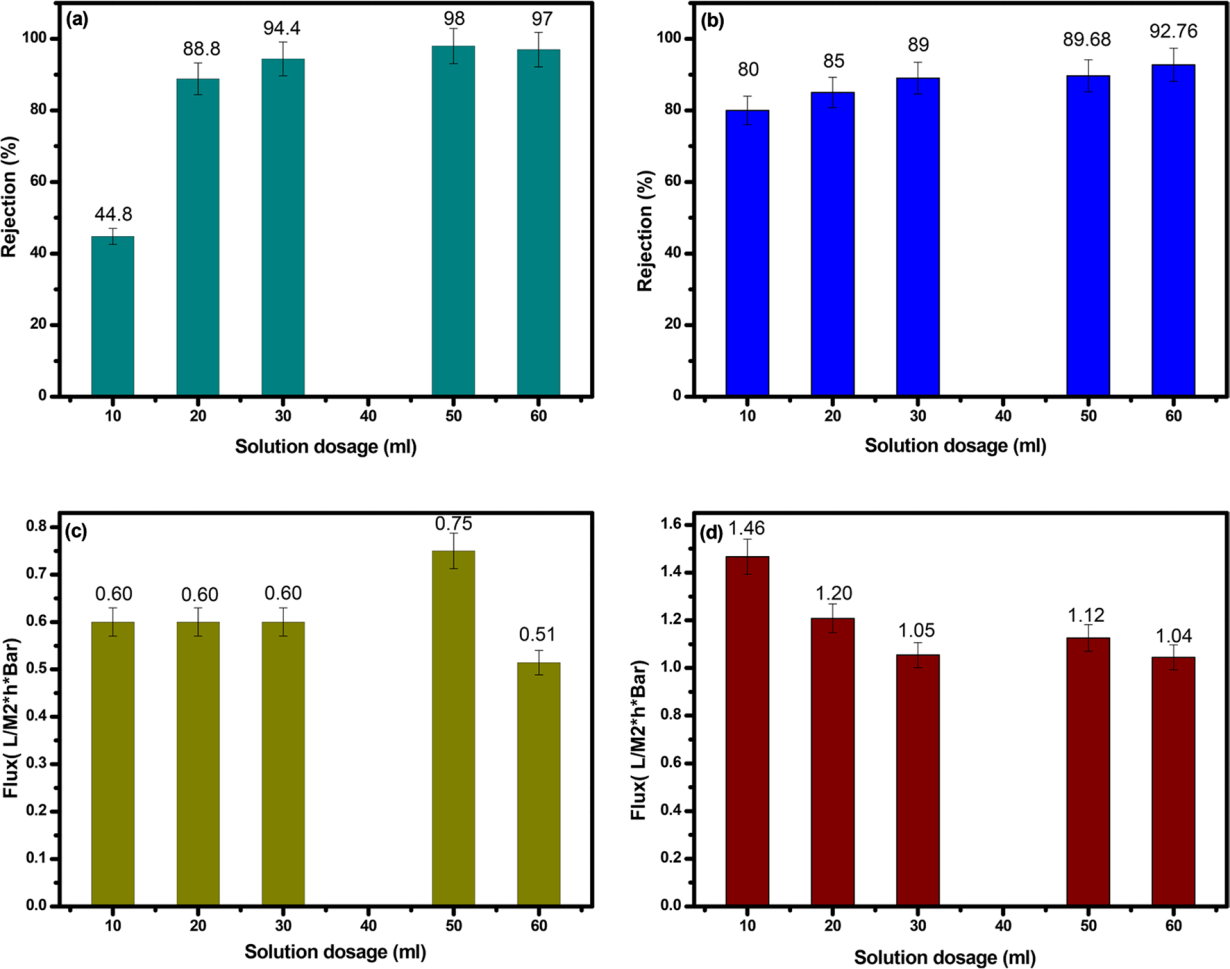
Effect of solution dosage on rejection rate of composite and blank membrane (**a** and **b**), and permeate flux (**c** and **d**), respectively

Initially, five membranes with dimensions of 0.2 m^2^, operating at 0.5 bar and with a solution concentration of 100 mg L^−1^, were fabricated using separated solutions containing sodium chloride (NaCl), humic acid (HA), and methylene blue (MB) at varying solution dosages. The results revealed that as the quantity of the solution increased, the separation performance of the PMC membrane improved, while the membrane flux decreased. Particularly, the PMC membrane exhibited an optimized separation efficiency of 98% and a flux of 0.514 at a solution dosage of 50 mL, while the PVC blank membrane achieved a separation efficiency of about 92.76% and a flux of 1.04 as exhibited in Fig. 5a, b and Fig. 5c, d, respectively. The observed increase in rejection for the PMC membrane with increasing solution dosage can be attributed to the incorporation of MC nanoparticles, which offer a high surface area and additional adsorption active sites for solutes. This enhancement in surface area and the improved interaction between the nanoparticles and solutes contribute to the increased rejection of the PMC membrane. Additionally, the presence of MC nanomaterials in the PMC membrane enhances its hydrophilicity, resulting in the generation of a thin water layer on the membrane’s surface, which effectively reduces fouling and enhances permeability.

#### Influence of solution concentration

**Fig. 6:**
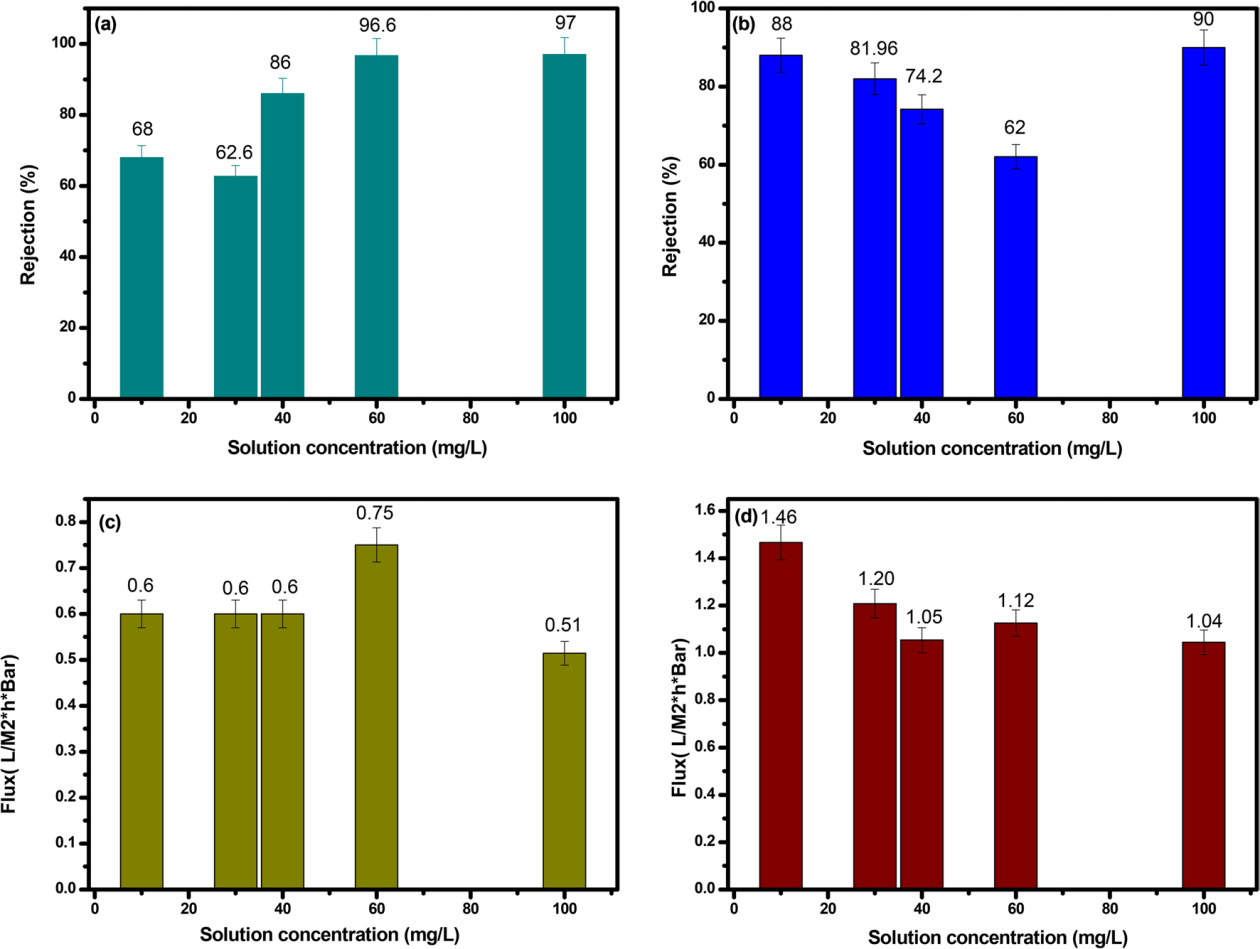
Impact of concentration dosage of on rejection rate of composite and blank membrane (**a** and **b**), and permeate flux (**c** and **d**) respectively

The effect of solution concentration on the PMC membrane’s performance was evaluated using five membranes (0.2 m^2^, 0.5 bar, and 50 mL) fabricated with 10 mL of solutions at different concentrations (10, 30, 40, 60, and 100 mg L^−1^). The results demonstrated that as the solution concentration increased, the separation efficiency of the PMC membrane improved, while the membrane flux decreased. At a solution concentration of 100 mg L^−1^, the PMC membrane achieved the highest separation efficiency of approximately 97% and a flux of 0.514, compared to the PVC blank membrane with a separation efficiency of about 90% and a flux of 1.04 as represented in Fig. 6a, b and Fig. 6c, d, respectively.

The increase in rejection observed in both the PMC membrane and PVC blank membrane with increasing solution concentration is attributed to the elevated driving force for diffusion across the membrane at higher solute concentrations. However, the presence of maghemite copper oxide nanoparticles in the PMC membrane results in higher rejection rates due to its enhanced selectivity. Moreover, the increased hydrophilicity of the PMC membrane facilitates higher permeability, leading to a lesser decline in flux compared to the PVC blank membrane with increasing solution concentration.

#### Pump pressure and flux relationship

**Fig. 7:**
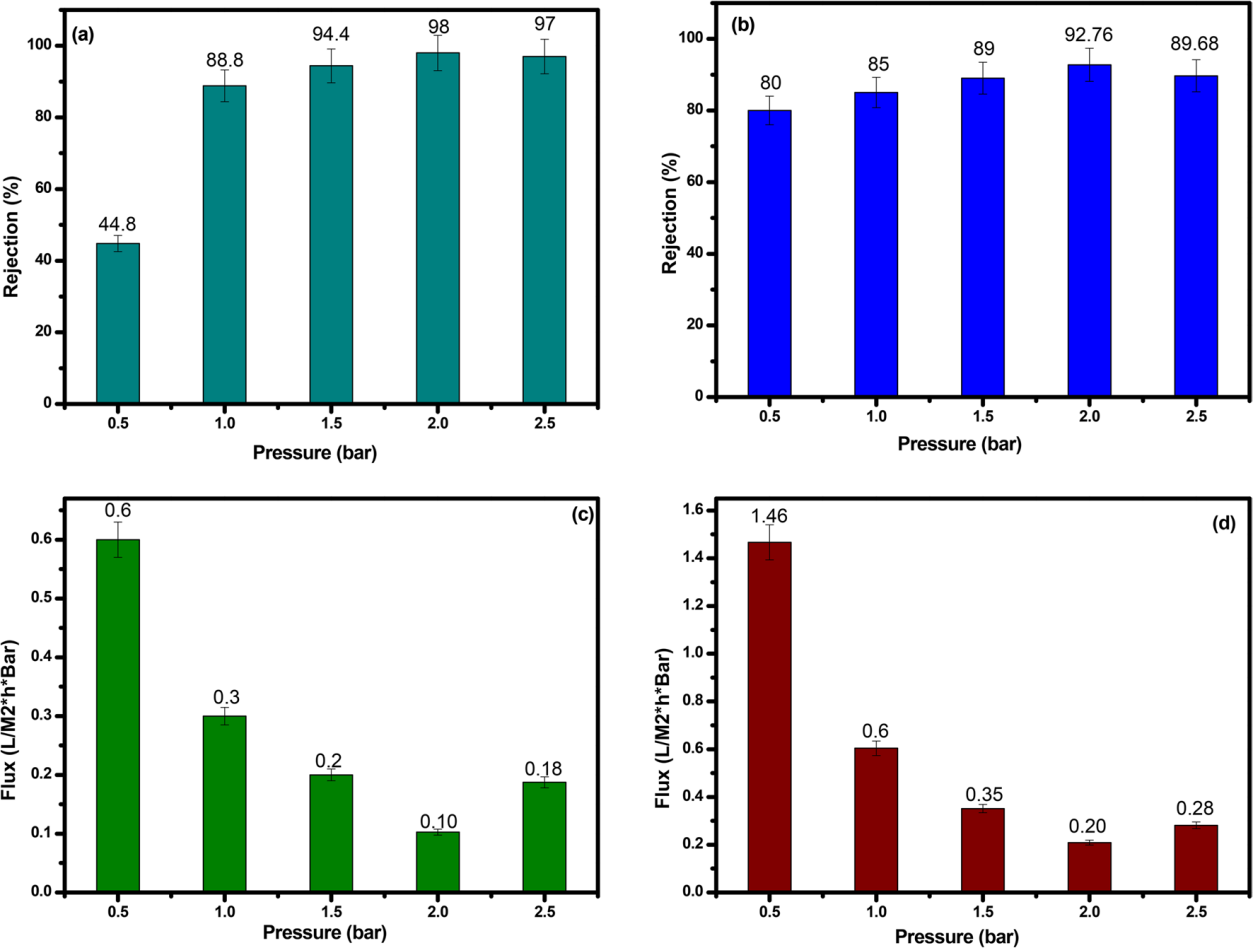
Effect of pressure on rejection rate of composite and blank membrane (**a** and **b**), and permeate flux (**c** and **d**) respectively

The impact of pump pressure on membrane flux was studied, and it was found that as pump pressure increased, the membrane flux also increased significantly, while the separation efficiency reduced slightly. At an optimized pressure of 2 bar, the PMC membrane exhibited a high separation efficacy of approximately 98% and a flux of 1.10, whereas the PVC blank membrane had a separation efficiency of about 92.79% and a flux of 0.20 as illustrated in Fig. 7a, b and Fig. 7c, d, respectively. The PMC membrane’s superior performance in flux is attributed to its optimized pore size, enabling it to maintain high permeability even at increased pump pressures. The incorporation of maghemite copper oxide nanoparticles further enhances the mechanical strength of the PMC membrane, allowing it to withstand higher pump pressures without compromising its performance.

The investigation of various operational parameters demonstrated the superior rejection and flux behavior of the PMC nanocomposite membrane compared to the PVC blank membrane. The presence of maghemite copper oxide nanoparticles contributed to its improved selectivity, hydrophilicity, and permeability, making it highly promising for water treatment applications. Furthermore, optimizing the operating conditions, including pressure, solution concentration, and dosage, is essential for maximizing the PMC membrane’s efficiency in real-world water treatment applications. It is important to note that the quantity of solutes in the feed solution plays a significant role in membrane performance, underscoring the importance of carefully tuning the operating parameters for optimal membrane efficiency. Additionally, further research is needed to address potential limitations such as fouling and to explore scalable fabrication techniques for broader implementation of the PMC membrane in water treatment processes.

### Contact angle measurement

Understanding the surface wettability of membranes is crucial for evaluating their performance in various separation processes, particularly in the context of oil-in-water emulsion separation. Contact angle measurements provide valuable insights into the interaction between a solid surface and different liquids, shedding light on the surface’s hydrophilic or hydrophobic nature. In this study, we conducted contact angle measurements to compare the wettability of composite membrane (PMC) and PVC membrane. This is done by the utilization of water, ethanol, and hexane, as three different test liquids, representing a spectrum of polarities. The contact angle, measured as the angle formed between the liquid droplet and the solid surface, serves as an indicator of the intermolecular forces between them. Lower contact angles typically signify higher surface wettability. The results of our contact angle measurements revealed significant differences between PMC and PVC membranes across all three test liquids. These differences hold vital implications for their performance in oil-in-water emulsion separation and related applications. PMC exhibited a contact angle of 65° when in contact with water, while PVC displayed a notably higher contact angle of 85°. This considerable contrast indicates that PMC possesses a substantially higher surface wettability when interacting with polar liquids like water. The reduced contact angle suggests that water readily spreads and adheres to the PMC membrane, which can be attributed to its intrinsic hydrophilic properties. When exposed to ethanol, PMC displayed a contact angle of 50°, which is considerably lower than the 65° contact angle observed for PVC. This result underscores PMC’s enhanced wettability with ethanol, another polar liquid. The reduced contact angle implies that ethanol interacts more favorably with the PMC membrane, which aligns with its superior hydrophilicity. In the case of hexane, a nonpolar solvent, PMC exhibited a contact angle of 25°, while PVC displayed a higher contact angle of 45°. This outcome suggests that PMC’s surface interacts more favorably with nonpolar liquids as well. The difference in contact angles with hexane may be indicative of variations in surface roughness or chemical properties between the two materials. PMC’s lower contact angle with hexane further supports its effectiveness in accommodating a wide range of liquids in separation processes. These wettability findings offer crucial insights into the superior performance of PMC in oil-in-water emulsion separation. The membrane’s enhanced surface wettability, particularly when in contact with polar liquids like water and ethanol, facilitates efficient removal of oil droplets from emulsions. Moreover, its favorable interaction with nonpolar solvents like hexane suggests its versatility and potential for various separation applications. So, based on all this, PMC outperforms PVC in terms of surface wettability. This characteristic plays a pivotal role in enhancing the efficiency and effectiveness of the PMC membrane in oil-in-water emulsion separation, contributing to its promising applications in addressing environmental and industrial challenges.

### Reusability

**Fig. 8:**
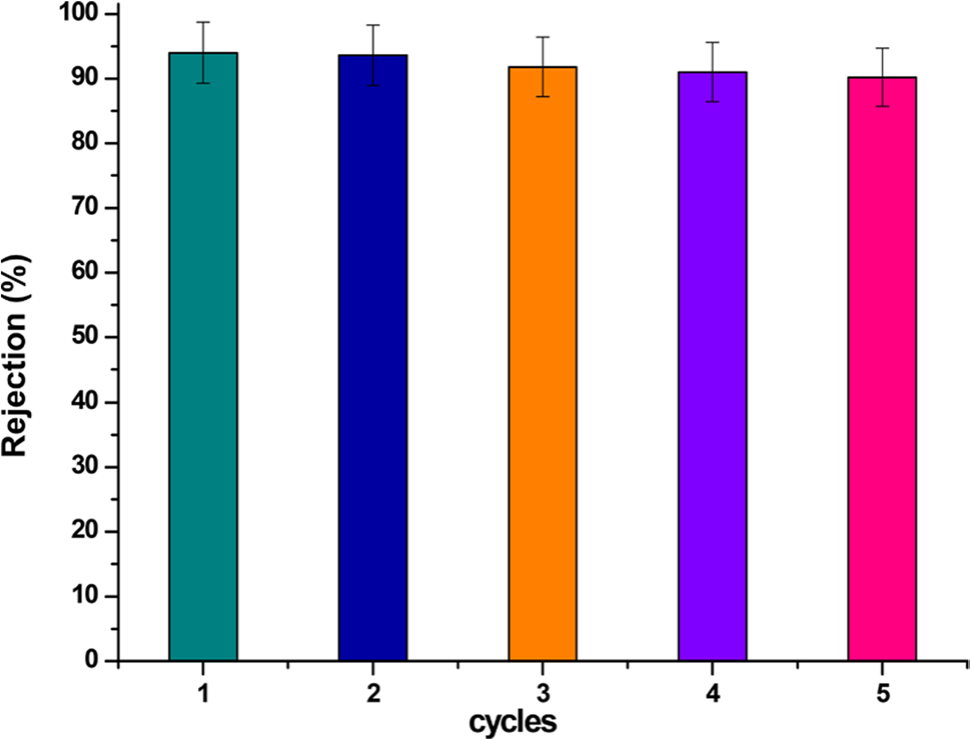
Reusability of PMC composite membrane for oil-in-water emulsions separation carried out at constant pressure of 2 bar (0.2 MPa)

The reusability of the modified membrane for oil-in-water emulsions separation was thoroughly evaluated in this study to assess its practical applicability and antifouling characteristics. After each cycle of emulsions separation, the composite membrane was carefully washed with hot water to remove any residual contaminants. Figure 8 illustrates the removal efficacy of oil-in-water emulsions over five repeated cycles. Remarkably, the removal efficiency of the composite membrane remained stable throughout the cycles, demonstrating consistent and reliable performance.

The composite membranes exhibited exceptional rejection capabilities, achieving an impressive removal efficiency ranging from 90.2 to 94% for oil-in-water emulsions. This represented a remarkable enhancement of up to 40% in solute removal compared to using a pure membrane alone. These findings validate the outstanding performance and efficiency of the modified composite membrane, positioning it as a highly promising solution for addressing oil-in-water emulsions separation challenges.

An intriguing aspect of the composite membrane’s behavior lies in its inherent antifouling properties (Liu et al. 2017; Makhetha and Moutloali 2018; Abedini 2019; Kotlhao et al. 2019; Shen et al. 2019; Nainar et al. 2020; Yang et al. 2020; Nwafor et al. 2021). The membrane’s ultrahydrophobicity and ultraoleophobicity in aqueous solutions, combined with its ultra-low oil adhesion properties, contribute to its remarkable stability and resilience against fouling (Yong et al. 2014; Crittenden et al. 2015; Rasouli et al. 2021; Zhao et al. 2021; Nayak et al. 2022). The ability to resist fouling is a critical advantage in practical applications, as it ensures consistent performance and prolongs the membrane’s operational life (Li and Chen 2010)(Alsawaftah et al. 2021). Furthermore, during the crossflow filtration process, we conducted extensive investigations to assess the stability of the asymmetric membrane. The results further confirmed the membrane’s robustness and its capability to withstand the rigors of filtration processes over an extended period, underlining its potential for industrial applications. To ascertain the composite membrane’s practical feasibility, it is important to consider potential challenges and limitations that may arise during real-world application. While the experimental results presented here are promising, further investigations under diverse operating conditions and with varying emulsions compositions would enrich our understanding of the membrane’s performance and highlight its versatility.

Overall, the reusability assessment of the modified maghemite copper oxide nanocomposite-based membrane has revealed its exceptional stability and antifouling characteristics for oil-in-water emulsions separation. The consistently high removal efficiency and enhanced solute rejection, combined with its resilience to fouling, underscore the membrane’s potential for diverse industrial applications (Sun et al. 2021). Continued research in this direction, addressing potential challenges and exploring wider operating conditions, will undoubtedly advance our understanding and implementation of this innovative membrane technology in various environmental and water treatment scenarios.

### Adsorpo-filtration mechanism of the maghemite copper oxide nanocomposite membrane

**Scheme 2:**
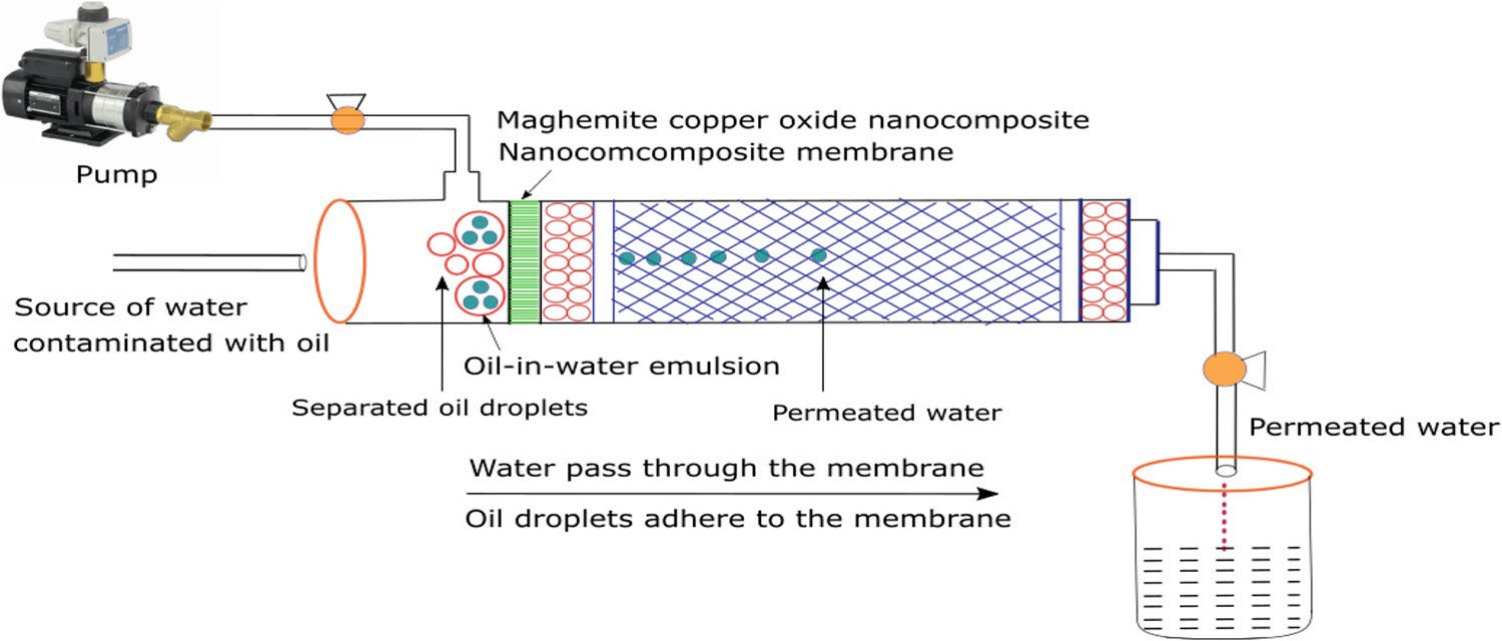
Illustration of oil-in-water emulsions separation through the nanocomposite membrane

Our in-depth exploration of the maghemite copper oxide (γ-Fe_2_O_3_/CuO) nanocomposite-based membrane reveals a highly sophisticated adsorpo-filtration mechanism that revolutionizes oil-in-water emulsion separation. This mechanism is a synergy of intricate physical and chemical processes that profoundly enhance membrane performance, making it a pivotal innovation in the field. At the heart of the adsorpo-filtration mechanism is the introduction of oil-in-water emulsions to the membrane’s surface. This pivotal step capitalizes on the membrane’s intrinsic hydrophobic nature, greatly expediting the adhesion of oil droplets to its surface. What is remarkable is that the incorporation of the maghemite copper oxide nanocomposite significantly augments the membrane’s hydrophobicity. This elevated hydrophobicity not only facilitates the adhesion of oil droplets but also ensures the swift passage of water through the membrane. The nanocomposite materials are characterized by high surface areas and generous pore volumes, thereby providing an optimal environment for the effective adsorption of oil droplets (Gawande et al. 2016; Abdullah et al. 2019; Govan 2020). A defining feature of the advanced adsorpo-filtration mechanism is the exceptional catalytic activity exhibited by the copper oxide nanoparticles within the nanocomposite (Gawande et al. 2016; Konar et al. 2016; Chavali and Nikolova 2019; Rabiee et al. 2020; Ning et al. 2022). As oil droplets adhere to the membrane’s surface, these nanoparticles actively participate in catalytic decomposition. This intricate process involves the breakdown of oil droplets into smaller, more manageable components. The catalytic decomposition acts as a powerful catalyst, significantly enhancing the overall efficiency of the separation process. This catalytic enhancement paves the way for subsequent steps to occur with remarkable ease. The adsorpo-filtration mechanism greatly amplifies the coalescence and separation process that takes place as oil droplets adhere to the membrane's surface. These droplets progressively merge, forming larger aggregates due to the attractive forces between the membrane and the oil (Tummons et al. 2016). This coalescence phenomenon plays a pivotal role in the separation mechanism. As oil droplets coalesce, they become too substantial to pass through the membrane’s pores, as vividly depicted in Scheme 2. This decisive outcome significantly enhances the separation efficiency, pushing it to unprecedented levels. The adsorpo-filtration mechanism is characterized by the harmonious synergy between physical processes, such as surface adsorption and coalescence, and the chemical process of catalytic decomposition facilitated by the copper oxide nanoparticles (Anandarup et al. 2016; Zedan et al. 2018; Ighalo et al. 2021). This unique synergy forms the backbone of the membrane’s capacity to attain exceptional separation efficiency. It presents a versatile and comprehensive solution for the separation of oil-in-water emulsions, effectively transcending the limitations of traditional separation methods. An integral aspect of this mechanism is its practicality and ease of maintenance. The membrane’s extraordinary characteristics, including ultra-hydrophobicity and ultra-oleophobicity, combined with its remarkable resistance to oil adhesion, contribute to its stability and resilience against fouling. What makes this mechanism even more appealing is its practical utility. The separated oil droplets can be effortlessly removed from the membrane’s surface with a simple washing using a suitable solvent. This practical feature ensures the membrane’s reusability and guarantees sustained high performance over multiple cycles of oil-in-water emulsions separation.

### Comparison with previous studies: enhancing oil–water separation performance

In the realm of enhancing oil–water separation performance through innovative membrane modifications, our maghemite copper oxide nanocomposite-based membrane emerges as a standout contender, a distinction underscored by a meticulous comparison with prior research. In an earlier investigation (Alghamdia et al. 2019), magnetite-modified membranes achieved an 89% oil rejection rate. However, our membrane’s ingenuity resides in the extraordinary amalgamation of maghemite copper oxide, rendering it ultra-hydrophobic and ultra-oleophobic. These characteristics synergistically bestow it with exceptional oil repellence and anti-fouling capabilities. Similarly, a prior study (Demirel et al. 2017) reported a 91.9% oil rejection rate for PVC/Fe_2_O_3_ membranes, yet our nanocomposite-PVC membrane surpassed expectations, reaching a remarkable 98% oil rejection rate. This advancement is attributed to the catalytic potency of copper oxide nanoparticles, enhancing oil droplet disintegration and separation. Delving deeper, another investigation (Marjani et al. 2020) explored the augmentation of polyethersulfone membranes through graphene oxide nanoparticles, achieving heightened water flux and salt rejection. Our nanocomposite-based membrane achieves analogous success, capitalizing on the unique attributes of maghemite copper oxide to realize enhanced rejection rates, minimizing fouling, and optimizing separation efficiency. Reflecting on a separate study (Cui et al. 2019), a PVDF@pDA@SiO_2_ nanocomposite membrane exhibited remarkable superhydrophilicity and antifouling attributes. Our maghemite copper oxide nanocomposite-based membrane ingeniously complements these qualities, integrating superhydrophobicity and catalytic activity, thereby elevating oil rejection rates and establishing a compelling edge in confronting intricate oil–water separation challenges. Looking ahead, despite an accomplishment of 93.4% separation rate through PVDF membranes modified with vermiculite nanoparticles in a recent study (Zhang et al. 2022), our membrane transcends this feat by achieving an impressive 98% maximum oil rejection rate, a feat underpinned by the strategic infusion of maghemite copper oxide, conferring exceptional oil-repelling aptitude. In a separate study, a newly engineered polyethersulfone (PES) membrane designed for the separation of oil-in-water emulsions exhibited an impressive separation efficiency of 90%. However, the PES membrane, despite its high rejection rate for larger oil droplets, was marked by restricted permeate flux and demonstrated low selectivity for smaller oil droplets. In sharp contrast, the PMC membrane excels in maintaining a superior equilibrium between separation efficiency and permeate flux. This characteristic positions the PMC membrane as a highly versatile choice suitable for a wide array of applications (Dmitrieva et al. 2022). In another research endeavor, scientists explored the utilization of a hybrid membrane composed of polyvinylidene fluoride (PVDF) and carbon nanotubes (CNTs) for the purpose of oil-in-water emulsion separation. While their membrane showcased an impressive separation efficiency exceeding 98%, it faced significant challenges in terms of mechanical strength and durability when subjected to demanding operational conditions (Wang et al. 2020).

Table 1 provides a comprehensive overview of various studies, including additional research, all of which collectively validate the remarkable effectiveness of our membrane in successfully separating emulsions within water. We duly recognize that multifaceted factors, spanning oil droplet dimensions, operational intricacies, and fabrication nuances, orchestrate membrane performance. However, the distinctive amalgam of ultra-hydrophobicity, ultra-oleophobicity, and catalytic efficacy distinctly positions our maghemite copper oxide nanocomposite-based membrane as a beacon of promise in the domain of advanced oil–water separation. Guided by foresight, forthcoming endeavors should pivot towards refining fabrication methodologies, navigating a spectrum of nanocomposite formulations, and comprehensively evaluating membrane performance across diverse operational contexts. These collaborative endeavors hold the potential to further heighten the potency and viability of our maghemite copper oxide nanocomposite-based membrane, ultimately advancing its mastery in tackling real-world oil–water separation complexities. In essence, this comparative exploration lucidly underscores the extraordinary achievements in oil rejection attained by our pioneering maghemite copper oxide nanocomposite-based membrane, heralding a new era of robust innovation in the realm of oil–water separation.

**Table 1:** Some previous studies reported membranes that effectively separate oil and water in emulsions

Membrane	Maximum oil rejection (%)	Reference
(PVC and Fe_3_O_4_) membrane	89	(Alghamdia et al. 2019)
(PVC and Fe_2_O_3_) membrane	91.9	(Demirel et al. 2017)
PVDF plant-derived epigallocatechin gallate (EGCG) with Ag	98.1	(Zhang et al. 2020)
PVC and acrylamide grafted bentonite (AAm-g-bentonite)	> 93.2	(Ahmad et al. 2023)
Diethylenetriaminepentaacetic acid-functionalized MWCNT/TiO2 (DTPA/MWCNT/TiO2)-PVDF membrane	97.4 ± 1	(Venkatesh et al. 2021)
Polysulfone/PEG	> 95	(Yuan et al. 2014)
Nitrile butadiene rubber (NBR) latex/(GO) membrane	92.23	(Abuhasel et al. 2021)
Ceramic (α-Al_2_O_3_- ZrO_2_)	> 90	(Milić et al. 2014)
(PVC and CuO and Fe_2_O_3_) membrane	98	This work

## Conclusion

In conclusion, we are pleased to introduce a groundbreaking maghemite copper oxide (MC) nanocomposite-based membrane meticulously designed to address the intricate challenge of separating oil from water emulsions. This remarkable membrane is characterized by its exceptional separation prowess, boasting a remarkable oil rejection rate of 98% and an ultra-high permeate flux of 0.102 L/m^2^ h. These achievements significantly surpass conventional pure PVC membranes, which typically exhibit a rejection rate of about 90% and an ultra-high flux of 0.085 L/m^2^ h. Our journey towards optimal membrane efficiency led us to unequivocally peak performance through a carefully orchestrated configuration, involving a solution dosage of 50 mL, a solution concentration of 100 mg L^−1^, and a pump pressure of 2 bar. These critical parameters have been meticulously fine-tuned to ensure that every facet of the membrane’s potential is fully harnessed. Our exploration extended further to reveal that the PMC nanocomposite membrane exhibited markedly lower contact angles (65° with water, 50° with ethanol, and 25° with hexane) compared to PVC membranes. This substantial reduction in contact angles, transitioning from 85 to 65° with water, 65 to 50° with ethanol, and 45 to 25° with hexane for pure PVC membranes, underscores the profound enhancement in hydrophilicity attributed to the heightened nanoparticle content. Moreover, we conducted an extensive structural and chemical analysis of the MC nanocomposite-based PVC membrane. Employing advanced techniques such as high-resolution transmission electron microscopy (HRTEM), scanning electron microscopy (SEM), X-ray diffraction (XRD), and Fourier transform infrared (FT-IR) spectroscopy, we intricately unveiled the structural and chemical attributes underpinning the membrane’s unparalleled performance. This research indeed marks a significant stride in oil–water separation technologies, promising a cleaner and more efficient pathway for environmental and water treatment processes. The extraordinary performance of the PMC nanocomposite-based membrane, coupled with its anti-fouling properties and enhanced efficiency, harbors immense potential across a multitude of industrial applications, from oil spill remediation to water purification and wastewater treatment. Looking forward, we envision exciting opportunities in this area for further research and exploration. These may encompass optimizing the nanocomposite composition, exploring different polymers for matrix fabrication, and delving into the underlying mechanisms of anti-fouling behavior. In conclusion, our study underscores the pioneering development of nanocomposite-based membranes, offering a transformative solution for the intricate challenge of oil-in-water emulsions separation. We hope our findings serve as an inspiring foundation for researchers and engineers to harness the vast potential of the PMC nanocomposite membrane, making a profound contribution to cleaner and more sustainable environmental practices. With its exceptional rejection rate, ultra-high flux, and the promise of further advancements, the maghemite copper oxide nanocomposite-based membrane is primed to revolutionize the landscape of oil–water separation technologies.

## Data Availability

All data underlying the results are available as part of the article and no additional source date are required.
